# Stone Fruit as Biofactories of Phytochemicals With Potential Roles in Human Nutrition and Health

**DOI:** 10.3389/fpls.2020.562252

**Published:** 2020-09-02

**Authors:** María Valeria Lara, Claudio Bonghi, Franco Famiani, Giannina Vizzotto, Robert P. Walker, María Fabiana Drincovich

**Affiliations:** ^1^ Centro de Estudios Fotosintéticos y Bioquímicos, Consejo Nacional de Investigaciones Científicas y Técnicas, Facultad de Ciencias Bioquímicas y Farmacéuticas, Universidad Nacional de Rosario, Rosario, Argentina; ^2^ Department of Agronomy, Food, Natural Resources, Animals and Environment, University of Padova Agripolis, Legnaro, Italy; ^3^ Dipartimento di Scienze Agrarie, Alimentari e Ambientali, Università degli Studi di Perugia, Perugia, Italy; ^4^ Department of Agricultural, Food, Environmental, and Animal Sciences, University of Udine, Udine, Italy

**Keywords:** chlorogenic acid, flavonoids, anthocyanins, carotenoids, postharvest, volatiles, health-promoting, cyanogenic compounds

## Abstract

Phytochemicals or secondary metabolites present in fruit are key components contributing to sensory attributes like aroma, taste, and color. In addition, these compounds improve human nutrition and health. Stone fruits are an important source of an array of secondary metabolites that may reduce the risk of different diseases. The first part of this review is dedicated to the description of the main secondary organic compounds found in plants which include (a) phenolic compounds, (b) terpenoids/isoprenoids, and (c) nitrogen or sulfur containing compounds, and their principal biosynthetic pathways and their regulation in stone fruit. Then, the type and levels of bioactive compounds in different stone fruits of the Rosaceae family such as peach (*Prunus persica*), plum (*P. domestica*, *P. salicina* and *P. cerasifera)*, sweet cherries (*P. avium*), almond kernels (*P. dulcis*, syn. *P. amygdalus*), and apricot (*P. armeniaca*) are presented. The last part of this review encompasses pre- and postharvest treatments affecting the phytochemical composition in stone fruit. Appropriate management of these factors during pre- and postharvest handling, along with further characterization of phytochemicals and the regulation of their synthesis in different cultivars, could help to increase the levels of these compounds, leading to the future improvement of stone fruit not only to enhance organoleptic characteristics but also to benefit human health.

## Health Promoting Properties of Fruit Phytochemicals

Here, a brief description of the main secondary organic compounds found in plants, their principal biosynthetic pathways, and their biosynthesis regulation in stone fruit is provided. Then, a review about the levels and types of secondary metabolites found in different stone fruit is presented. The factors that have been identified as being involved in defining the levels of these compounds in stone fruit are finally presented. It is concluded that the identification of the key regulatory points in the biosynthesis of these compounds or in their chemical modification to produce more compounds with better activity, as well as the identification of pre- and postharvest managements that could increase their levels, will aid in the future improvement of stone fruit for the benefit of human health.

Phytochemicals known as secondary metabolites possess diverse physiological properties, being involved in sensory attributes (aroma, taste and color) and in defense against pathogens, different kind of stresses and/or injuries (Tomas-Barberan et al., 2001; Montevecchi et al., 2013). Besides, the secondary metabolites of plants are highly beneficial to consumers. In this regard, in recent decades, consumers have become more aware of the relationship between diet and diseases. Today, there is a broad consensus that increased consumption of fruits and vegetables contributes to improving health and well-being by reducing the risk of diseases, such as cardiovascular diseases and some forms of cancer (Riboli and Norat, 2003; Hung et al., 2004). The Joint FAO/WHO (Food and Agriculture Organization/World Health Organization) report on diet, nutrition, and prevention of chronic diseases recommended in 2003 the intake of a minimum 400 g of fruits and vegetables per day, ideally 800 g, for the prevention of chronic diseases such as cancer, diabetes, heart disease and obesity (WHO Technical Report Series, 2003).

The health promoting properties of fruits and vegetables are due to the presence of some vitamins (such as A, C, E, and folates), dietary fibers, and secondary metabolites, some of which are unique of plants. Phenolic compounds, among which flavonoids are the most ingested during daily life from products of plant origin (Chun et al., 2007), are important secondary organic metabolites in terms of their health-promoting properties, with possible preventive role in neurological disorders and potential protection against chronic diseases (Weng and Yen, 2012; Silva and Poganik, 2020). In addition, different biological activities have been described for chlorogenic acid, a phenolic compound, which include anti-inflammatory (Liang and Kitts, 2016), anticancer (Liu Y. J. et al., 2013), antioxidant power, including the inhibition of lipid oxidation (Sasaki et al., 2010), antilipidic, antiepileptic, neuroprotective (Aseervatham et al., 2016), antidiabetic (Meng et al., 2013), and antihypertensive (Suzuki et al., 2006; Zhao et al., 2012). This phenolic compound has also beneficial effects in disorders related to the metabolic syndrome (Bhandarkar et al., 2019a; Bhandarkar et al., 2019b). Moreover, it possesses antimicrobial activity against a wide range of organisms, including bacteria, yeasts, molds, viruses, and amoebas, and thus, it could be used as an antimicrobial agent (Santana-Gálvez et al., 2017) for the preservation of food products. In addition, anthocyanins, another group of polyphenols, prevent tumor development by inhibiting cancer cells proliferation when tested *in vitro* and *in vivo*. Anthocyanins also exhibit anti-inflammatory activity, present neuroprotective, anti-obesity, antidiabetic activities and are proposed to prevent cardiovascular disease (extensively reviewed in Li et al., 2017). With respect to caroteonids, their role in human nutrition is well established as precursors of vitamin A and due to their antioxidant activity. Their protective function in liver health is well documented and reviewed by Elvira-Torales et al. (2019). Other metabolites that have received attention are the cyanogenic glycosides, which possess anticancer properties (Fukuda et al., 2003, Jaswal et al., 2018).

Different studies have indicated that stone fruits are particularly rich in important phytochemicals, which constitute an extra benefit to their pleasant taste and flavor. These metabolites include phenolic compounds and terpenoids, among others. Peach, plums, cherries have an important antioxidant activity due to their phenolic content (Kim et al., 2003b; Cevallos-Casals et al., 2006; Serra et al., 2011). *In vitro* studies have demonstrated the antimicrobial activity of plum and peach extracts (Cevallos-Casals et al., 2006; Belhadj et al., 2016). *In vitro* and *ex vivo* studies also showed the anti-inflammatory properties of *P. persica* extracts (Gasparotto et al., 2014). Pharmacological studies showed that *P. persica* has antihypertensive properties (Kim et al., 2019), and it influences the central cholinergic system (Kim et al., 2003c). Moreover, intake of peaches protects rat tissues from nicotine toxicity (Kim et al., 2017). Almond intake reduces cardiovascular disease risk by modulating plasma lipoproteins (extensively reviewed in Berryman et al., 2011), contributes to satiety (Hull et al., 2015), delays lipid bioaccessibility (reviewed in Grundy et al., 2016), and decreases inflammation and oxidative stress (reviewed in Kamil and Chen, 2012). Apricot has also an important therapeutic and nutritional value. Among its health promoting activities, antimicrobial, antimutagenic, cardio-protective, hepato-protective, and antioxidant properties have been described (Ozturk et al., 2009; Erdogan-Orhan and Kartal, 2011; Yurt and Celik, 2011; Chen et al., 2020).

## Main Secondary Organic Compounds Found in Plants: General Description of the Biosynthetic Pathways and Regulation with Emphasis in Stone Fruits

**Figure 1:**
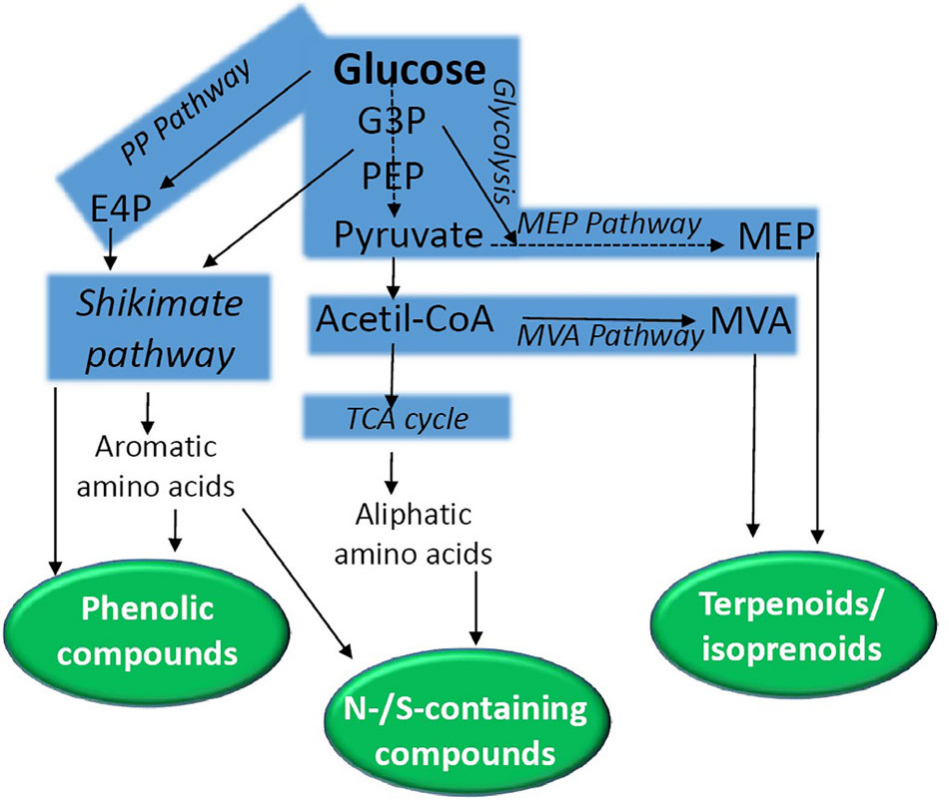
A simplified scheme of the biochemical pathways conducting the synthesis of secondary metabolites in plants. Natural products are grouped into phenolic compounds, N-/S-containing compounds, and terpenoids. E4P, erythrose 4-phosphate; G3P, glyceraldehyde 3-phosphate; MEP, 2-*C*-methyl-d-erythritol 4-phosphate; MEV, mevalonate; PP, Pentose phosphate; PEP, phosphoenolpyruvate; TCA, tricarboxylic acid.

Secondary metabolites can be grouped into three major classes: (a) phenolic compounds, (b) terpenoids/isoprenoids, and (c) nitrogen or sulfur containing compounds. These phytochemicals are derived from main primary pathways (glycolysis, the tricarboxylic citric acid (TCA) cycle, the pentose phosphate pathway, aliphatic and aromatic amino acids and the shikimic acid pathway) (**Figure 1**) (Aharoni and Galili, 2011). The shikimate pathway is a key route that conducts the synthesis of tyrosine, phenylalanine, and tryptophane (for review, see Tzin and Galili, 2010; Vogt, 2010; Maeda and Dudareva, 2012). This synthetic route starting from phosphoenolpyruvate (PEP) and erythrose 4-phosphate (E4P) connects primary metabolism to aromatic amino acid biosynthesis. This pathway, together with its intermediate metabolites (*i.e.* chorismate), provides precursors for the biosynthesis of folates, quinones, phytohormones, alkaloids, indole glucosinolates, flavonoids, hydroxycinnamic acids, lignins, and lignans.

### Phenolic Compounds

Phenolic compounds are synthesized from shikimic/phenylpropanoid and the phenylpropanoid–acetate–malonate pathways and encompass a large group of monomeric and polymeric phenols and polyphenols. Phenylalanine is the precursor of a wide range of volatiles including phenylpropenes, phenylpropanes, phenethyl derivatives, and benzenoids (Gonda et al., 2018).

**Figure 2:**
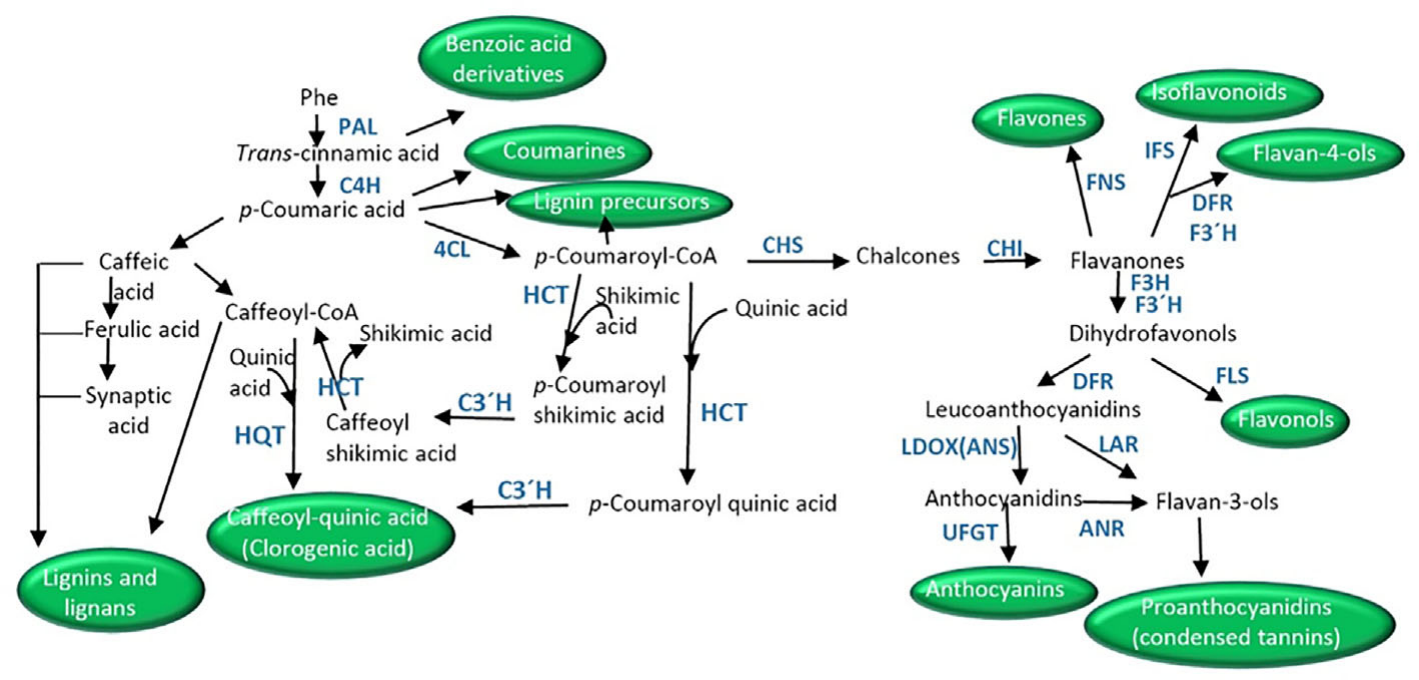
Schematic of biosynthetic pathways derived from phenyalnaline leading to the synthesis of coumarins, benzoic acid derivatives, clorogenic acid, lignans, lignins, and the different flavonoids (isoflavonoids, flavonols, flavones, flavonls, anthocyanins, proanthocyanidins). ANR, anthocyanidin reductase; CHI, chalcone isomerase; CHS, chalcone synthase; C4H, cinnamate-4-hydoxylase; C3′H, *p*-coumarate 3′-hydroxylase; 4CL, *p*-coumaroyl CoA ligase; DRF, dihydroflavonol 4-reductase; F3H, flavanone 3-hydroxylase; FNS, flavone synthase; F3′H, flavonoid 3′-hydroxylase; FLS, flavonol synthase; HCT, hydroxycinnamoyl-CoA shikimate/quinate hydroxycinnamoyl transferase; HQT, hydroxycinnamoyl-CoA quinate hydroxycinnamoyl transferase; IFS, isoflavone synthase; LDOX/ANS, leucoanthocyanidin dioxygenase/anthocyanidin synthase; LAR, leucoanthocyanidin reductase; PAL, phenylalanine ammonia lyase; UFGT, UDP-flavonoid glucosyl transferase.

Phenylpropanoids are simple phenolic compounds with a benzene ring and a lateral chain, which serve as precursors of compounds such as benzoic acid derivatives, flavonoids, coumarins, stilbenes, lignans and lignins, and condensed tannins (Oksana et al., 2012). The phenylpropanoid pathway initiates with phenylalanine synthesized in the shikimic acid pathway, which generates cinnamate by the action of phenylalanine ammonia lyase (PAL, **Figure 2**). The consecutive action of cinnamic acid 4-hydroxylase (C4H) renders 4-coumarate that finally gives 4-coumaroyl-CoA by the action of 4-coumarate-CoA ligase (4CL).

Phenolic acids include derivatives of benzoic acid (C_6_–C_1_) such as hydroxybenzoic acids and of cinnamic acid (called hydroxycinnamic acids, C_6_–C_3_) (Walker and Famiani, 2018). The hydroxybenzoic acids 4-hydroxybenzoic acid (4-HBA), vanillic acid (3-methoxy-4-hydroxy) and protocatechuic acid (3,4-dihydroxy) are constituents of lignin (Pietta et al., 2003). On the other hand, gallic acid is present in hydrolysable and condensed tannins. Hydroxycinammic acids include caffeic (3,4-dihydroxycinnamic acid), ferulic (4-hydroxy-3-methoxycinnamic acid), sinapic acids (3,5-dimethoxy-4-hydroxy) and *p*-coumaric (4-hydroxy) acid, and their conjugates, mainly as esters of quinic acid (chlorogenic acids).

#### Chlorogenic Acid

Chlorogenic acid (5-O-caffeoylquinic acid) is an ester of caffeic acid and (−)-quinic acid from the hydroxycinnamic acid family. It can be found in different foods and herbs (Santana-Gálvez et al., 2017; Walker and Famiani, 2018).

Early studies proposed the direct synthesis of this compound through transesterification from caffeoyl-CoA (synthesized from caffeic acid in the reaction catalyzed by *p*-coumarate 3-hydroxylase, C3H) and quinic acid by the action of hydroxycinnamoyl-CoA quinate hydroxycinnamoyl transferase (HQT, Stöckigt and Zenk, 1974, **Figure 2**). Nevertheless, the main route in higher plants is the 3′-hydroxylation of *p*-coumaroyl quinic acid by the cytochrome P450 monooxygenase *p*-coumarate 3′-hydroxylase or *p*-coumaroyl ester 3′-hydroxylase (C3′H, Niggeweg et al., 2004; Abdulrazzak et al., 2006; **Figure 2**). *p*-Coumaroyl quinic acid is synthesized from *p*-coumaroyl-CoA and quinic acid by the activities of a hydroxycinnamoyl-CoA shikimate/quinate hydroxycinnamoyl transferase (HCT). In addition, the action of C3′H on *p*-coumaroyl shikimate (synthesized from *p*-coumaroyl-CoA and shikimate by HCT) to give caffeoyl shikimic, which then by the action of HCT (Hoffmann et al., 2003) gives shikimic acid and caffeoyl-CoA has also been described. Caffeoyl-CoA then is the substrate of HQT in the first reaction (**Figure 2**).

#### Flavonoids

Flavonoids consist of a fifteen-C phenylpropanoid core (the flavan skeleton) formed by two aromatic rings connected by a heterocyclic pyran ring (C_6_–C_3_–C_6_). Depending on the oxidation and number of unsaturations of the pyran ring, flavonoids are classified into different groups named as flavones, flavanones, isoflavones, flavonols, 3-deoxy flavonoids, and anthocyanins (Andersen and Markham, 2005).

The general pathway of flavonoid synthesis branches from the phenylpropanoid and malonic pathways (**Figures 1** and **2**). The first committed step is catalyzed by the enzyme chalcone synthases (CHS) which synthesizes 4,2′,4′,6′-tetrahydroxychalcone (chalcone) by condensing *p*-coumaroyl-CoA with malonyl-CoA (Jez and Noel, 2000). Chalcones are the main precursors of all flavonoids (Dixon and Steele, 1999). The product of CHS is later isomerized into flavanones (*e.g.*, naringenin or eriodictyol) by chalcone isomerase (CHI) (Winkel-Shirley, 2001). Flavanones are the precursors of flavones in the reaction catalyzed by flavone synthase (FNS) (Martens and Mithofer, 2005). Alternatively, flavanones give rise to dihydroflavonols (dihydrokaempferol), precursors of anthocyanins and proanthocyanidins, by the action of flavanone 3-hydroxylase (F3H). Dihydroflavanols can be routed to flavonols’ synthesis by the action of flavonol synthase (FLS) (Pelletier et al., 1997). On the other hand, the action of flavonoid 3′-hydroxylase (F3′H) or flavonoid 3′5′-hydroxylase (F3′5′H) transforms dihydrokaempferol into dihydroquercetin or dihydromyricetin, respectively.

The existence of large number of flavonoids is the consequence of the different modifications on the main compounds by the action of different glycosyltransferases, methyltransferases, and acyltransferases (Saito et al., 2013). Flavonoid aglycones can be glycosylated at positions C-3, C-5, and C-7. The ability to glycosylate flavonol, anthocyanidin, and anthocyanin aglycones depends on the type of glycosyltransferase (Saito et al., 2013). Instead, flavonoid methyltransferases (FMTs) methylate flavonols giving isorhamnetin (Tohge et al., 2007).

Enzymes of the flavonoid biosynthetic pathway are grouped forming metabolons channelizing the different intermediates into the different routes (Winkel-Shirley, 1999). Although being cytosolic, the enzymes of the flavonoid pathway are bound to the cytoplasmic face of the endoplasmic reticulum by interacting with cytochrome P450 proteins (Saslowsky and Winkel, 2001). In addition, enzymes of the pathway such as CHS, CHI (Saslowsky et al., 2005), and FLS (Kuhn et al., 2011) have also been found in the nucleus.

#### Anthocyanins

Anthocyanins are water-soluble pigments belonging to the flavonoids and one of the main compounds responsible for coloration in plants (Tanaka et al., 2008). Their accumulation in the vacuole gives red, orange, blue, and purple color to different plant tissues and organs (Grotewold, 2006). Fruit color is a key quality trait, in which anthocyanin accumulation is involved.

Anthocyanins are synthesized as part of the flavonoid pathway through the action of dihydroflavonol 4-reductase (DFR, Heller et al., 1985; Reddy et al., 1987, **Figure 2**). The enzyme converts the dihydroflavonols or eriodictyol, a flavanone, to leucoanthocyanidins. The next step is catalysis by the action of a leucoanthocyanidin dioxygenase/anthocyanidin synthase (LDOX/ANS) which finally gives the anthocyanidins (Nakajima et al., 2001). A great number of anthocyanidins have been described in plants, of which delphinidin, pelargonidin, cyaniding, and luteolinidin are the most abundant. Anthocyanidins are the precursors of 2,3-*cis*-2R,3R-flavan 3-ols (known as proanthocyanidins or condensed tannins; *i.e.* epicatechin) by the reaction catalyzed by anthocyanidin reductase (ANR, Xie et al., 2003). Condensed tannins contribute to the astringent flavor of fruits. Leucocyandins can conduct the synthesis of catechin, another proanthocyanidin, by the action of leucoanthocyanidin reductase (LAR). Chatechins are the units of the polymeric proanthocyanidins constituting a subgroup of flavonoids. Chatechins can be galloylated by esterification with gallate in the 3-position of the C-ring (He et al., 2009). Alternatively, anthocyanidins can be modified by glycosylation to yield different anthocyanins (**Figure 2**). UDP-flavonoid glucosyl transferases (UFGT) catalyze the glycosylation of flavonoids using a UDP-sugar at 3-, 5-, 7-, 3′-, or 4′-OH positions (Tohge et al., 2005; Bowles et al., 2006; Pang et al., 2008; Yonekura-Sakakibara et al., 2009; Montefiori et al., 2011). In addition, acylation and methylation also increase anthocyanin variability (Nakayama et al., 2003; Matsuba et al., 2010; Miyahara et al., 2012; Miyahara et al., 2013). Anthocyanin modification increases anthocyanin stability and water solubility due to intramolecular and/or intermolecular stacking (Springob et al., 2003).

Anthocyanin synthesis occurs in the cytosol through the flavonoid biosynthetic enzymes associated with the cytoplasmic face of the endoplasmic reticulum (Winkel-Shirley, 1999). Once formed, anthocyanins are stored in the vacuole to prevent oxidation (Marrs et al., 1995). Glutathione S-transferases (GSTs), transporters, and vesicles are involved in their transport into the vacuole (Grotewold, 2004). Depending on the species, anthocyanins are found uniformly distributed inside the vacuole or accumulated in discrete sub-vacuolar structures (Grotewold and Davis, 2008) anthocyanoplasts, intravacuolar pigmented globules’ (cyanoplasts, Nozue et al., 1993) or anthocyanic vacuolar inclusions (Markham et al., 2000).

The anthocyanin biosynthetic pathway is transcriptionally controlled by MYB and basic helix–loop–helix (bHLH) transcription factors, together with the WD40 proteins characterized by seven regions of 40 amino acids rich in tryptophan and aspartic acid (Quattrocchio et al., 1999; Hernandez et al., 2004). The ternary complex regulates the steps of the pathway in a spatial and temporal way during plant development (Hichri et al., 2011), and it is called MBW complex (Gonzales et al., 2008). bHLH and WD40 act as co-activators of MYBs, which may repress or activate anthocyanin biosynthetic genes (Koes et al., 2005; Dubos et al., 2008). bHLH proteins are essential to enhance the MYB-induced anthocyanin synthesis. NAC proteins are also involved in the regulation of anthocyanin synthesis in *Arabidopsis* (Morishita et al., 2009). Different factors such as light, temperature, and hormones modulate anthocyanin synthesis (Jeong et al., 2004; Takos et al., 2006; Steyn et al., 2009; Zhang et al., 2012). Anthocyanin biosynthesis is also developmentally controlled as demonstrated by the variation of promoter methylation of *MdMYB10*, the master regulator of the anthocyanin pathway in apple (El-Sharkawy et al., 2015).

Studies about the regulation of the biosynthesis of some secondary compounds have been started to emerge in the Rosaceae family (Lin-Wang et al., 2010). In Japanese plum (*Prunus salicina*), RNA seq analysis allowed the identification of genes involved in anthocyanin synthesis. In the late stages of fruit maturation, when anthocyanin content increases, the expression of several genes, such as *PsPAL*, *PsC4H*, *Ps4CL*, *PsCHS*, *PsCHI*, *PsF3H*, *PsF3*′*H*, *PsDFR*, *PsANS*/*LDOX*, *PsUFGT*, and *PsGST*, is increased. Fang et al. (2016) found that *MYBs* expression correlated with structural genes. While c39005.graph_c0 (homologous to AtMYB113) positively correlated with anthocyanin biosynthetic genes, c29499.graph_c0 (homologous to AtMYB73) and c32850.graph_c0 (homologous to AtMYB102) showed a negative correlation. With respect to bHLH transcription factors, 36695.graph_c0 (homologous to AtTT8) positively correlated with anthocyanin contents, and 33382.graph_c0 (homologous to AtbHLH14) decreased with ripening. In addition, a gene encoding a NAC (c27539.graph_c0, AtNAC100) was also up-regulated and positively correlated with the expression of genes involved in anthocyanin synthesis (Fang et al., 2016).

In prunus, levels of anthocyanins are responsible for the color and vary depending on the genotype, external factors, and organs. Moreover, Cheng et al. (2015) revealed that *PsPAL, PsCHS, PsCHI, PsF3H, PsDFR, PsLDOX*, and *PsUFGT* are up-regulated by ethylene treatment and repressed by 1-MCP. They showed that *PsMYB10* is involved in this regulation. More recently, Niu et al. (2017) demonstrated that at high temperatures, anthocyanin levels are regulated not only by the synthesis but also by their degradation. Furthermore, they showed that hydrogen peroxide triggers anthocyanin enzymatic degradation *via* catalysis by a vacuolar peroxidase. Furthermore, Cheng et al. (2014) showed the expression of *PpUGT78A1, PpUGT78A2, PpUGT78B*, and *PpUGT79B*, encoding flavonoid 3-O-glycosyltransferase, varies among tissues and development, conducting the different patterns of anthocyanin accumulation.

Different studies were conducted to get insight into anthocyanin level regulation exploring the eventual redundancy of TFs during peach ripening (Lin-Wang et al., 2010; Ravaglia et al., 2013; Rahim et al., 2014). Analysis of MYB10 sequences reveals that R2R3 sequences from Rosaceae are highly conserved (Lin-Wang et al., 2010). Of the six MYB10s detected in peach genome (Verde et al., 2013) only *PpMYB10.1-3* is expressed in the fruit (Rahim et al., 2014). Transient luciferase assays in *Nicotiana benthamiana* probed that *PpMYB10.2* from peach (as well as those from pear, European plum, cherry-plum, cherry and apricot, denominated *PcMYB10, PdmMYB10, PcfMYB10, PavMYB10, ParMYB10*, and *PprMYB10*, respectively) induced anthocyanin synthesis activating Arabidopsis DFR-promoter when co-expressed with a bHLH (Lin-Wang et al., 2010). In addition, in the presence of bHLH, PpMYB10.2 also transactivated a reporter gene driven by a peach *UFGT* promoter (Ravaglia et al., 2013). *PpMYB10.1* and *PpMYB10.3* were shown to correlate with anthocyanin content in the peel, mesocarp, and mesocarp around the stone of peach fruit and with anthocyanin structural genes CHS, F3H, DFR and UFGT (Rahim et al., 2014). According to Tuan et al. (2015) *PpMYB10.1* is a key factor regulating anthocyanin levels in red-skinned peach (Japanese peach cultivar ‘Akatsuki’) and that it activates *PpUFGT* transcription. They proposed that *PpMYB10.2/*3 could have a different role than pigment regulation since in ‘Mochizuki’ and ‘Akatsuki’ cultivars the transcript levels of *PpMYB10.2* and -3 do not correlate with anthocyanin accumulation. Moreover, *PpMYB10.2* was expressed in leaves that do not exhibit anthocyanin accumulation. In addition, Zhou et al. (2014) also indicated that PpMYB10.2 is not involved in anthocyanin synthesis in leaves. On the other hand, *PpMYB10.4* is expressed in leaves, and it controls anthocyanin accumulation in this organ. *PpMYB10.4* is mapped within the interval of the red allele that controls peach leaf color (Zhou et al., 2014). Studies in flowers reveal that *PpMYB9*, is an activator highly expressed in peach flowers and it regulates UFGT gene expression. In the presence of PpbHLH3, PpMYB9 is able to induce anthocyanin synthesis when expressed in tobacco leaves. It is proposed that the *PpMYB9* genes have diverged in functions from the MYB10 genes (Zhou et al., 2016). PpMYB17-20 are repressors, which levels varied during the different stages of flower development (Zhou et al., 2016).

Among three different bHLH candidate genes (based on homology with Arabidopsis), *PpbHLH3* allowed the induction of the pigments when *PpMYB10.1 or* *PpMYB10.3* were expressed in tobacco. Analysis of the genome reveals that *PpMYB10.1-3* are located within 80 kb on pseudomolecule 3, within the two closest markers (CC2 and CC12A) to the *anther color* trait, and thus highlighting the role of these transcription factors in the anthocyanin synthesis regulation (Rahim et al., 2014). On the other hand, Zhou et al. (2015b) analyzing the segregation of the blood-flesh trait attribute (due to anthocyanin accumulation) in a peach landrace Dahongpao showed that *PpMYB10.1* did not co-segregate with this trait (Zhou et al., 2015b). However, by heterologous expression in tobacco, they found that BLOOD (BL), a NAC transcription factor, and PpNAC1 act together to transactivate the *PpMYB10.1* gene. *PpSPL1*, a SQUAMOSA promoter-binding protein-like transcription factor (SPL), represses this transactivation. Another R2R3-MYB gene regulating PA synthesis in peach is *PpMYB7*, which activates the transcription of *PpLAR1* but not *PpANR. PpMYB7* promoter has an ABA-dependent DRE2 element and it can be activated by the basic leucine-zipper 5 TF PpbZIP5 *via* the ABA signaling (Zhou et al., 2015a).

In peach, anthocyanin accumulation is also influenced by light quality (including UV-B, UV-A, blue light and (far) red light) and depends on the genetic background (Liu T. et al., 2015). The expression of bHLH3 also correlated with induction of anthocyanin synthesis in peach peel under UV exposure (Zhao et al., 2017). Light effect is likely mediated by PpHYH that is also homologous to *A. thaliana* HY5, rather than by *PpHY5* (Zhao et al., 2017). In addition, ppa009438m, ppa009380m, ppa026582m, ppa007883m, and ppa025263m encoding PpNACs responded to UV-B light. Nevertheless, *BL* and *PpNAC1* did not respond to UV-B treatment in ‘Hujingmilu’ and ‘Yulu’ cultivars (Zhao et al., 2017). Therefore, the levels of anthocyanin in peach fruit are complexly controlled by the coordination of a set of transcription factors.

Other transcription factors such as PpMYBPA1 (ppa009439m) and Peace (ppa023768m) that is phylogenetically related to PpMYBPA1, have been identified in RNAseq studies and related to the regulation of proanthocyanin (PA) biosynthesis in peach fruit (Wang et al., 2013). *PpMYBPA1*, as in the case of *PpMYB7*, can be activated by PpbZIP5 *via* the ABA signaling (Zhou et al., 2015a). In flowers, PpMYBPA1 and Peace were to be expressed at bud stage and were shown to activate *PpLAR* and *PpANR* promoters (Zhou et al., 2016).

While many advances have been conducted to elucidate the transcription factors controlling anthocyanin accumulation in peach, less information is available with respect to apricot. Some cultivars have blushed skin due to the presence of cyanidin-3-O-glucoside, cyanidin-3-O-rutinoside, and peonidin-3-O-rutinoside. Thus, anthocyanin presence and accumulation depend on the cultivar. In apricot, *PaMYB10* also correlated with anthocyanin accumulation in blushed apricots and with *PaMYB10, PaPAL, PaCHS, PaCHI, PaF3H, PaDFR, PaLDOX*, and *PaUFGT*. The overexpression of this MYB transcription factor in fruits of Luntaixiaobaixing cultivar conducted the red coloration of the skin. *PaMYB10* was found to be located with Linkage group 3 (G3) related to skin color (García-Gómez et al., 2019). In addition, it was probed that anthocyanin accumulation in apricot was also influenced by light, as fruit bagging affects the coloration of the skin (Xi et al., 2019). Sugars were also correlated with the content of cyanidin-3-O-glucoside and cyanidin-3-O-rutinoside; as it the case of sorbitol, glucose, fructose and sucrose (Huang et al., 2019). Further studies are required to decipher the way in which sugars affect anthocyanin synthesis in apricot.

In sweet cherry, the expression of *PacCHS, PacCHI, PacF3H, PacDFR, PacANS, and PacUFGT* correlated with anthocyanin accumulation (Liu et al., 2013). The regulation of this pathway has started to emerge. Sequencing of cDNAs encoding enzymes involved in the synthesis of anthocyanins or their regulators (bHLH and WD40) confirms the conservation at sequence level within the Prunus genus (Starkevič et al., 2015). Up to now, *PavMYB10, PavMYBA*, and *PavMYB1* have been studied in sweet cherries (Lin-Wang et al., 2010; Shen et al., 2014; Starkevič et al., 2015; Jin et al., 2016). The first transcription factor modulating the pathway characterized was PavMYB10, which was probed to correlate with anthocyanin accumulation in fruit (Lin-Wang et al., 2010). Two sub-variants of *PaMY10.1, PaMYB10.1-1* and *-3*, were later studied. By studying fruits at different stages of development from different cultivars of *P. avium*, Starkevič et al. (2015) indicated that *PaMYB10.1-1* parallels that of anthocyanin accumulation and that *PaMYB10.1-3* is expressed at low levels in fruit. Nevertheless, when transiently expressed in tobacco, *PaMYB10.1-3* was able to induce pigment synthesis. It was also shown that PavMYB10.1 has three alleles *PavMYB10.1a-to -c*. These alleles are responsible for the variation in color of the fruit. *PavMYB10.1a* regulates the expression of *PavANS* and *PavUFGT* by binding with *PavbHLH* and *PavWD40* (Jin et al., 2016). Jin et al., 2016 proposed that PavMYB10.1 is a DNA molecular marker for the color of the skin.

With respect to bHLHs, *PabHLH3 and -33* are expressed in fruit. While PabHLH3 co-activates anthocyanin biosynthesis in the presence of MYB, PabHLH33 acts as a co-repressor (Starkevič et al., 2015). In addition, *PavMYBA*, encoding for a R2R3-MYB TF from red-colored sweet cherry, was studied. Transient assays demonstrated that this factor binds to bHLHs and activates the expression of *PavDFR, PavANS*, and *PavUFGT*. In addition, ABA modulated the synthesis of anthocyanins in cherry fruit, with *PavMBA* involved in this process (Shen et al., 2014).

### Terpenoids

The terpenoids or isoprenoids constitute a large group of metabolites derived from C_5_ isoprene (2-methyl-1,3-butadiene) formed by “head-to-tail” conjugation. Terpenoids fulfill varied biological functions in plants, from essential roles as electron transport chain components, pigments (carotenoids, and chlorophylls), elements of membrane structure and function (phytosterols), hormones (*i.e.* gibberellins, strigolactones, brassinosteroids, abscisic acid, isopronoids cytokinins), protein glycosylation (dolichols), to defense (antimicrobial/anti-insect) and attractants (volatile signals) (Tholl, 2015; Tarkowská and Strnad, 2018; Block et al., 2019). Volatile terpenoids (*i.e.* R-limonene) are the major class of volatile compounds in plants and are predominantly isoprenes, mono- and sesquiterpenes. Volatile terpenoids have been found in roots, stems, leaves, fruits, seeds and to higher extent in flowers. In fruits, these terpenoids contribute to aroma production (Dudareva et al., 2013; Abbas et al., 2017).

**Figure 3:**
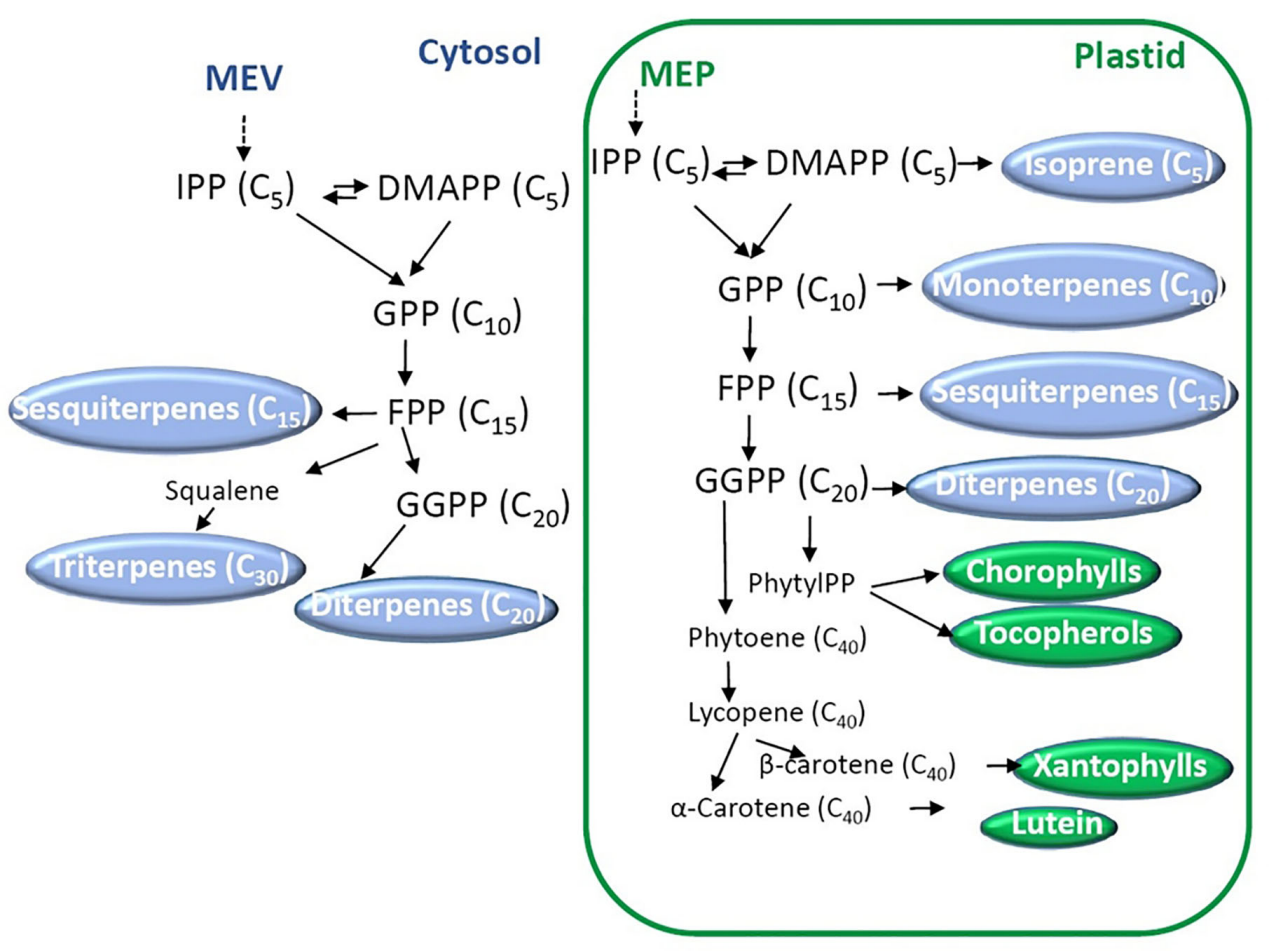
Biosynthesis of terpenoids. Two main pathways starting from mevalonate (MEP) and 2-*C*-methyl-d-erythritol 4-phosphate (MEP) occur in the cytosol and the plastids, respectively. Dimethylallyl diphosphate (DMAPP) and its isomer isopentenyl diphosphate (IPP) are the isoprene units that conduct the synthesis of the building blocks geranyl diphosphate (GPP), farnesyl diphosphate (FPP), and geranylgeranyl diphosphate (GGPP). The number of carbons of each molecule is indicated between brackets.

Biosynthesis and functions of isoprenoids have been well characterized and extensively reviewed (Tholl, 2015; Yazaki et al., 2017; Tarkowská and Strnad, 2018). According to their number of units they can be divided into mono- (C_10_), sesqui- (C_15_), di- (C_20_), sester- (C_25_), tri- (C_30_), tetra- (C_40_, carotenoids), and polyterpenes (>C_40_) (Tholl, 2015). The combination of isoprene units, in the form of dimethylallyl diphosphate (DMAPP) and its isomer isopentenyl diphosphate (IPP), conducts the synthesis of the building blocks geranyl diphosphate (GPP), farnesyl diphosphate (FPP) and geranylgeranyl diphosphate (GGPP, **Figure 3**). These precursors are synthesized by two distinct pathways: the mevalonate (MVA) and the nonmevalonate pathways. The last route is also called the 2-*C*-methyl-d-erythritol 4-phosphate (MEP) or the 1-deoxy-d-xylulose 5-phosphate pathway (DOXP). MVA derives from mevalonate synthesized from acetyl-CoA, while MEP derives from pyruvate. GPP, FP, and GGPP are the precursors in the synthesis of mono-, sesqui- and diterpenes, respectively (Tholl, 2015). Then, terpene synthases (TPSs) and cytochrome P450s are the main enzymes that generate the huge terpenoid diversification using mono-, sesqui-, and diterpenes. Other enzymes also contribute to the array of chemically diverse terpenoids.

To mention an example of biosynthesis, carotenoids are synthesized from GGPP using phytoene synthase (PSY) which condenses two molecules of GGPP to render 15-cis phytoene. The following enzymes in the route are phytoene desaturase (PDS), 15-cis-ζ-carotene isomerase (ZISO), ζ-carotene desaturase (ZDS), and carotenoid isomerase (CRTISO) giving *trans*-lycopene (Liang et al., 2018). Lycopene is the precursor of many molecules of biological importance, the *δ*-, *γ*-, *α*-, *ϵ*-, and *β*-carotenes. Carotenes are also important precursors; lutein and xanthopylls derive from *α*- and *β*-carotene, respectively (for further details, see Liang et al. (2018)). Apocarotenoids are the result of the oxidative cleavage of carotenoids. Examples of apocarotenoids are the derivatives of *β*-ionone that have pleasant scent and aroma in fruits and flowers (Hou et al., 2016). While many efforts have been dedicated to the elucidation of carotenoid metabolism in plant species, little is known about the regulation (Pott et al., 2019).

Regulation of carotenogenesis has been exhaustively investigated in tomato fruit and Arabidopsis, and it has been also comprehensively reviewed (Llorente et al., 2017; Stanley and Yuan, 2019). Transcription factors such as phytochrome interacting factors (PIFs), ethylene response factors (ERFs), MADS ripening inhibitor factor (RIN) are some of the many TFs that modulate carotenoids synthesis. Both environmental conditions (*i.e.* light) and developmental cues control the carotenoid biosynthetic genes (Llorente et al., 2016; Sun et al., 2018). The control of carotenoid accumulation is also exerted at posttranscriptional and posttranslational levels (Stanley and Yuan, 2019). Biosynthesis, degradation, and storage mediate carotenoids homeostasis as well (Li and Yuan, 2013).

The first study of regulation of carotenoids’ synthesis regulation in apricot was conducted comparing varieties with contrasting colors. It showed similar regulation of the synthesis of the colorless carotenoids phytoene and phytofluene due to ethylene upregulation of PSY1 and PDS. In this study, while great differences in *β*-carotene levels were found in two varieties, accumulation of ZDS was found in both varieties (Marty et al., 2005). Further, RNAseq analysis revealed that structural genes like PDS1, carotenoid cleavage dioxygenase (CCD)1 and 4, violaxanthin de-epoxidase 1 (VDE1) and zeaxanthin epoxidase (ZEP) are the main regulatory points for carotenogenesis (Zhang et al., 2019). Network analysis indicates the presence of two modules for gene expression for carotenoids synthesis in apricots. A set of transcription factors integrating hormones such as ethylene and brassinosteroids (ERF4/5/12, AP2, AP2-like and BZR1), developmental factors (MADS14, NAC2/25, MYB1R1/44, GLK1/2 and WRKY6/31/69) and light (PIF3/4 and HY5) possibly modulate carotenoid accumulation during ripening (Zhang et al., 2019).

In peach, studies regarding carotenoid accumulation in the mesocarp have been focused on carotenoid cleavage. CCDs control carotenoid degradation, and therefore color accumulation. *PpCCD4* encodes a carotenoid dioxygenase. High transcript levels increase carotenoid degradation producing colorless compounds and thus, rendering white flesh. *PpCCD4* is also associated with synthesis of carotenoid-derived volatiles (Brandi et al., 2011; Adami et al., 2013; Falchi et al., 2013). In the mentioned studies and in Cao et al. (2017), although carotenoid synthesis is linked to their biosynthetic enzymes, correlation between biosynthetic genes’ expression and carotenoid levels was not found. What is clear is that the levels of *PpCCD4* increase in the flesh and the pulp of white varieties during development and ripening. Yellow varieties are characterized by a combination of higher levels of *PpPDS* and lower accumulation of *PpCCD4* with respect to the white ones (Cao et al., 2017). In addition, while blue light treatment applied during storage was effective to stimulate the transcription of carotenoid biosynthetic genes in both yellow and white varieties, it was able to induce carotenoid accumulation only in yellow but not in white peach, indicating that in white peach the levels of main carotenoid biosynthetic genes are not the primary factor regulating carotenoid amounts (Cao et al., 2017).

### Nitrogen- and Sulfur-Containing Secondary Metabolites

The third group of secondary metabolites is large and diverse. It includes alkaloids, glucosinolates, and cyanogenic glucosides.

Glucosinolates are nitrogen- and sulfur-containing metabolites generally restricted to the *Brassicales* (Grubb and Abel, 2006). Glucosinolates are diverse and their derivatives participate in plant defense and contribute to flavor and aroma (Grubb and Abel, 2006).

Alkaloids are nitrogenous compounds of low molecular weight derived mostly from amino acids (Ziegler and Facchini, 2008). This is a diverse group with a wide range of biological functions. According to their structure, alkaloids can be classified into “true” alkaloids with a heterocyclic nitrogen, protoalkaloids (no cyclic nitrogen), and pseudoalkaloids (*e.g.*, steroidal and diterpene alkaloids, caffeine) (Waterman, 1998). The different biosynthetic pathways are in accord to their diversity in structure (Ziegler and Facchini, 2008).

Cyanogenic glycosides participate in plant defense and are widely distributed in the plant kingdom (Zagrobelny et al., 2008). The basic structure is a carbon backbone derived from an amino acid and a glycosilated cyanohydrin. The participation in plant defense mechanisms relays on the hydrolytic release of hydrocyanic acid potentially toxic to herbivores (HCN) (Drochioiu et al., 2008). The occurrence of cyanogenic glycosides has been described in species of the Fabaceae, Poaceae, Rosaceae, and Asteraceae, and also in conifers and ferns (Gleadow and Møller, 2014). Seeds from stone fruits of the Rosaceae family are rich in diglucoside (R)-amygdalin (Swain et al., 1992). In addition to kernels, the monoglucoside prunasin is also found in leaves, roots, stems (Dicenta et al., 2002; Sánchez-Pérez et al., 2008). Both compounds are synthesized from phenylalanine. The sequential action of two cytochrome P450 enzymes belonging to the CYP79 and CYP71 families catalyze the synthesis of mandelonitrile (Yamaguchi et al., 2014), which is then glucosylated by a UDP-glycosyltransferase (UGT) to form prunasin. This compound is then glucosylated again to form amygdalin.

In *Prunus dulcis*, two genes *PdCYP79D16* and *PdCYP71AN24* encoding CYP79 and 71 and three genes encoding UGTs involved in amygdalin (*PdUGT94AF1* and *PdUGT94AF2*) and prunasin synthesis (*PdUGT94AF3*) were identified. The functionality and activities of the proteins were tested by expression in *Nicotiana benthamiana*. Comparisons of sweet and bitter almonds, which differentially accumulate amygdalin, indicated that the expression of *PdCYP79D16* and *PdCYP71AN24* is very low or null in the tegument of the sweet genotype and high in the bitter almonds (Thodberg et al., 2018). More recently, using map-based cloning and a segregating population for sweet kernel trait Sánchez-Pérez and colleagues (2019) identified that bHLH2 controls the expression of *PdCYP79D16* and *PdCYP71AN24.* A mutation in bHLH2 avoids the transcription of both genes, and thus mandelonitrile, amygdalin and prunasin are not synthesized. Selection of this mutation is proposed to have allowed the domestication of almond (Sánchez-Pérez et al., 2019).

## Stone Fruits as a Source of Phytochemicals: A Description Of Secondary Organic Compounds’ Composition of Main Stone Fruits

### Peach

Phenolic compounds are the main source of antioxidant capacity in peaches (Gil et al., 2002). They also participate in the visual appearance (pigmentation and browning) (Lee et al., 1990) and in the taste (astringency) of fruits (Tomás-Barberán et al., 2001) and therefore could be used to evaluate the quality of the fruit. In peaches, carotenoids are responsible for the yellow color of the pulp, and therefore, their concentration is low in white pulp fruits (Gil et al., 2002). Peaches contain anthocyanins (cyanidin-3-glucoside and cyanidin-3-rutinoside), flavan-3-ols (catechin—the main monomeric flavan-3-ol-, epicatechin, epigallocatechin, and procyanidins), flavonols (quercetin-3-O-rutinoside or rutin, quercetin-3-glucoside, quercetin-3-galactoside, kaempferol-3-rutinoside), hydroxycinnamic acids (chlorogenic and neoclorogenic acids) (Campbell and Padilla-Zakour, 2013; Dabbou et al., 2017), and the main carotenoids include *β*-carotene and xanthophylls (mono- or di-hydroxylated carotenoids), zeaxanthin, *β*-cryptoxanthin and violaxanthin (Tomas-Barberan et al., 2001; Campbell and Padilla-Zakour, 2013; Dabbou et al., 2017), and lutein (Oliveira et al., 2016). Amounts of some of the most representative phytochemicals present in the pulp of peach of different cultivars at commercial maturity are shown in **Table 1**.

**Table 1:** Amounts of some of the most representative phytochemicals present in the pulp of peach, plum, apricot, and cherry at commercial maturity.

	*Prunus persica*		*Prunus salicina*		*Prunus armeniaca*		*Prunus avium*	
Neochlorogenic acid	Annongshuimi	2.6^c^	Keckemetska ruza	1.19^l^	Early Magic	18.1^i^	Burlat	21.7^s^
	Huyou0 002	23.2^c^	Madjarska najbolja	1.42^l^	Beltsville Elite B70197	215.4^i^	Saco	190^s^
	Huyou0 018	7.8^c^	Velika rana	1.22^l^	NY101	179.4^i^	Summit	40.4^s^
	Sweet cap	5.8^d^	Bebecou	1.37^m^	Brite pearl	183^j^	Badascony	4.74^t^
	Ealy May Crest	5.0^d^	Nafsika	0.41^m^	September red	24.1^j^	Early Van Comact	11.9^t^
	O’Henry	4.1^d^	Niove	1.57^m^	Spring Bright	36.7^j^	Vigred	6.50^t^
	Big Top	2.3^e^	Z 109/58	3.8^n^	Laetitia	39.6^k^	Della Marca	0.96^u^
	Royal Glory	1.5^e^	Rojo passion	10.68^n^	Ruby Red	40.1^k^	Lapins	6.17^u^
	Red Haven	4.7^e^	Z 505/2	6.4^n^	African delight	39.1^k^	Moretta	7.31^u^
Chlorogenic acid	Annongshuimi	4.1^c^	Keckemetska ruza	1.47^l^	Brite pearl	277^j^	Burlat	3.65^s^
	Huyou0 002	31.1^c^	Madjarska najbolja	2.29^l^	September red	39^j^	Saco	12^s^
	Huyou0 018	23.3^c^	Velika rana	1.93^l^	Spring Bright	84.3^j^	Summit	9.73^s^
	Sweet cap	8.9^d^	Bebecou	1.01^m^	Early Magic	0.9^i^	Badascony	1.12^t^
	Ealy May Crest	8.5^d^	Nafsika	0.46^m^	Beltsville Elite B70197	9.5^i^	Early Van Comact	2.26^t^
	O’Henry	5.6^d^	Niove	2.2^m^	NY101	21.0^i^	Vigred	1.07^t^
	Big Top	0.21^e^	Z 109/58	6.9^n^	Laetitia	NDk	Della Marca	22.83^u^
	Royal Glory	0.44^e^	Rojo passion	11.0^n^	Ruby Red	ND^k^	Lapins	149.89^u^
	Red Haven	0.35^e^	Z 505/2	7.6^n^	African delight	2.5^k^	Moretta	68.4^u^
Rutin	Annongshuimi	ND^c^	Bebecou	9.37^m^	Brite pearl	27.2*	Burlat	3.06^s^
	Huyou0 002	ND^c^	Nafsika	5.65^m^	September red	41.8*^j^	Saco	13.69^s^
	Huyou0 018	ND^c^	Niove	7.73^m^	Spring Bright	56.9*^j^	Summit	2.81^s^
	Sweet cap	0.25^d^	‘Keckemetska ruza	2.02^l^	Early Magic	6.3^i^	Badascony	5.78^t^
	Ealy May Crest	0.13^d^	Madjarska najbolja	1.55^l^	Beltsville Elite B70197	4.3^i^	Early Van Comact	3.53^t^
	O’Henry	0.14^d^	Velika rana	2.17^l^	NY101	5.0^i^	Vigred	5.68^t^
	Big Top	1.8^e^	LE-2927	12.16*	Laetitia	6.6^k^	Della Marca	5.13^u^
	Royal Glory	0.99^e^	Salah-Jerevan	5.41°	Ruby Red	7.9^k^	Lapins	51.97^u^
	Red Haven	0.8^e^	Chuan Zhi Hong	56.9°	African delight	7.4^k^	Moretta	41.4^u^
Cyanidin-3- rutinoside	Annongshuimi	3.5^c$^	Z 115/26	1.6^n^	Early Magic	18.9^i^	Burlat	28.5^s^
	Huyou0 002	1.3^c$^	Rojo passion	4.4^n^	Beltsville Elite B70197	25.7^i^	Saco	24.5^s^
	Huyou0 018	3.8^c$^	Z 505/2	2.9^n^	Longjohn	33.0^i^	Summit	20.1^s^
	Sweet cap	0.028^d^			Brite pearl	3.5*^j^	Badascony	12.8^t^
	Ealy May Crest	0.011^d^			September red	4.6*^j^	Early Van Comact	8.06^t^
	O’Henry	1.148^d^			Spring Bright	12.7*^j^	Vigred	13.5^t^
	Big Top	0.47^e$^			Laetitia	1.48^k^	Della Marca	2.05^u^
	Royal Glory	0.67^e$^			Ruby Red	2.81^k^	Lapins	389.9^u^
	Red Haven	0.42^e$^			African delight	1.51^k^	Moretta	268.2^u^

The amounts of chlorogenic and neochlorogenic acids (hydroxycinnamic acids), quercetin-3-O-rutinoside or rutin (flavonol) and cyanidin-3-rutinoside (anthocyanins) are indicated in different cultivars and expressed in mg/100 g of fresh weight.

^c^ Zhao et al., 2015; ^d^ Dabbou et al., 2017; ^e^ Manganaris et al., 2017; ^i^ Kim et al., 2003a; ^j^ Tomas-Barberan et al., 2001; ^k^ Venter et al., 2013; ^l^ Dragovic-Uzelac et al., 2007; ^m^ Roussos et al., 2011; ^n^ Ruiz et al., 2005; °Schmitzer et al., 2011; ^s^ Goncàlves et al., 2004; ^t^ Usenik et al., 2008; ^u^ Martini et al., 2017.

More than 100 volatile compounds have been identified in peach fruit. Volatile organic compounds define fruit aroma and participate, together with organic acids and sugars, in fruit taste. Aroma is a central trait that influences the fruit quality perception by consumers (Bruhn et al., 1991). In addition, volatile composition gives significant information regarding healthful composition of food since they are synthesized from essential nutrients (Goff and Klee, 2006). Peach volatiles have been classified into alcohols, aldehydes, carboxylic acids, non-cyclic esters, terpenoids, ketones, and lactones (Wang et al., 2009; Sánchez et al., 2012). The most abundant are C_6_ compounds, esters, benzaldehyde, linalool, C1_3_ norisoprenoids, and lactones. The amounts of benzaldehyde, linalool, *δ*-decalactone and (E)-2-hexenal in four peach cultivars are presented in **Table 2** to show their variability among cultivars.

**Table 2:** The most abundant or representative volatiles (benzaldehyde, linalool, trans-linalool oxide, *γ*-decalactone, *δ*-decalactone, hexanal, (E)-2-hexenal, hexanol, 2-Hexen-1-ol and hexyl acetate) in the pulp of different cultivars of peach, plum, apricot, and cherry at commercial maturity are expressed in µg/kg of fresh weight.

Prunus persica		
2-Hexenal	Western red	1,381.1^a^
	Chongyanghong	2,332^&b^
	Zaohongxia	3,228^&b^
	Wuyuehuo	7078^&b^
Benzaldehyde	Western red	61.9^a^
	Chongyanghong	1,187.8^&b^
	Zaohongxia	2,918.4^&b^
	Wuyuehuo	1,080.4^&b^
Linalool	Western red	50.2^a^
	Chongyanghong	285.9^&b^
	Zaohongxia	680.2^&b^
	Wuyuehuo	759.3^&b^
*δ*-decalactone	Western red	16.3^a^
	Chongyanghong	1,904.5^&b^
	Zaohongxia	1,368.5^&b^
	Wuyuehuo	1,843.0^&b^
***Prunus armeniaca***		
(E)-2-Hexenal	Palstey	4,635^p^
	Moniqui	26,800^p^
	Rouge du Roussillo	8,752^p^
	Early Blush Rutbhart	133^q^
	Spring Blush EA3126TH	59^q^
	PBS 28-58	153^q^
	K604-19	ND^r^
	K113-40	ND^r^
	K33-81	ND^r^
Linalool	Palstey	3,019^p^
	Moniqui	1,021^p^
	Rouge du Roussillo	864^p^
	Early Blush Rutbhart	80^q^
	Spring Blush EA3126TH	256^q^
	PBS 28-58	43^q^
	K604-19	671^r^
	K113-40	365^r^
	K33-81	150^r^
*δ*-decalactone	Palstey	2526^p^
	Moniqui	37,310^p^
	Rouge du Roussillo	21,024^p^
	Early Blush Rutbhart	454^q^
	Spring Blush EA3126TH	105^q^
	PBS 28-58	281^q^
	K604-19	1,424^r^
	K113-40	3^r^
	K33-81	50r
Hexyl acetate	Palstey	5,244^p^
	Moniqui	1,250^p^
	Rouge du Roussillo	13,140^p^
	Early Blush Rutbhart	54^q^
	Spring Blush EA3126TH	2^q^
	PBS 28-58	3^q^
	K604-19	ND^r^
	K113-40	ND^r^
	K33-81	4^r^
***Prunus salicina***		
2-Hexenal	Horvin	72^g^
	Range	1.03^h^
1-Hexanol	Showtime	861^f^
	Laetitia	1,370^f^
	Primetime	1210^f^
	Horvin	1,739.7^g^
	Range	2.11^h^
Hexanal	Showtime	1,500^f^
	Laetitia	1,850^f^
	Primetime	2,420^f^
	Horvin	Traces^g^
	Range	7.67^h^
trans-Linalool oxide	Primetime	185^f^
	Laetitia	262^f^
	Showtime	726^f^
	Horvin	102.2^g^
***Prunus avium***		
(E)-2-	Van	412.71^v^
Hexenal	Vista	45.53^v^
	0-900Ziraat	269.50^v^
	Canada	2.56^w^
	Ferrovia	6.36^w^
	Lapins	2.60^w^
Benzaldehyde	Van	10.10^v^
	Vista	7.69^v^
	0-900Ziraat	20.52^v^
	Canada Giant	0.45^w^
	Ferrovia	0.50^w^
	Lapins	1.06^w^
Hexanal	Van	122.96^v^
	Vista	25.75^v^
	0-900Ziraat	144.66^v^
	Canada Giant	0.71^w^
	Ferrovia	2.72^w^
	Lapins	0.62^w^
2-Hexen-1-ol	Van	172^v^
	Vista	14.64^v^
	0-900Ziraat	83.77^v^
	Canada Giant	1.02^w^
	Ferrovia	1.04^w^
	Lapins	0.78^w^

^&^the ripening stage is not specified in the literature; ND, not detected; ^a^ Aubert et al., 2014; ^b^ Zhu and Xiao, 2019; ^f^ Cuevas et al., 2016; ^g^ Pino and Quijano, 2012; ^h^ Chai et al., 2012; ^p^ Guichard and Souty, 1988; ^q^ Aubert and Chanforan, 2007; ^r^ Gómez et al., 1993; ^v^ Hayaloglu and Demir, 2015; ^w^ Vavoura et al., 2015.

Lactones such as *γ*-decalactone, c-jasmolactone c-octalactone, c-dodecalactone, *δ*-decalactone and 6-pentyl-*α*-pyrone have been described as the key odorants to the pleasant aroma of peach fruit, with *γ*-decalactone most highly associated with the “peach-like” note. In addition, the esters (Z)-3-hexenyl acetate, (E)-2-hexen-1-ol acetate, and ethyl acetate add to the “fruity” notes. Moreover, linalool and *β*-ionone and other terpenoids contribute to the “floral” notes (Horvat et al., 1990; Derail et al., 1999; Eduardo et al., 2010).

During industrial processing of peach to produce juices, marmalades, concentrates and canned fruit, peel and stone are removed and discarded. Likewise, most of the times that fruit is freshly consumed; the peel is also discarded because of the use of chemicals and pest contamination or digestion problems. Nevertheless, both peels and kernels are a source of bioactive compounds. Indeed, many phytochemicals are more abundant in the fruit peel than in the edible fleshy parts. For example, phenolics (Tomás-Barberán et al., 2001), carotenoids, and ascorbic acid double their amount in the peel than in the pulp (Gil et al., 2002). Dabbou et al. (2017) showed that irrespective of the ripening stage, hydroxycinnamic acids, total flavonols, and total anthocyanins are higher in the peel than in the pulp. In contrast, the relative content of carotenoids depended on the harvesting stage.

Peach kernels provide seed oil and fatty acids (Wu et al., 2011). In addition, kernels are a source of a wide range of metabolites of nutritional importance. The following secondary metabolites have been identified in peach kernels: protocatechuic acid, *p*-hydroxybenzoic acid, *p*-hydroxyphenylacetic acid, dihydroxybenzoic acid, chlorogenic acid, *p*-coumaric acid, ferulic acids, dithiothreitol, rutin, caffeic acid, procyanidin B2, hydrocinnamic acid, procatechol, catechin, gentisic acid, kuromanin chloride, vanillic acid, epicatechin gallate, sinapinic acid, and ellagic acid (Wu et al., 2011 and Koprivica et al., 2018), with catechin being prevalent.

Much effort has been dedicated to the characterization of the volatilome of peach as well as the profiling of other secondary metabolites in stone fruits (Horvat et al., 1990; Visai and Vanoli, 1997; Derail et al., 1999; Eduardo et al., 2010; Sánchez et al., 2012). In addition, in the case of volatiles, some QTLs have been identified (Sánchez et al., 2014).

### Apricot

Apricot is a good source of carotenoids (Zaghdoudi et al., 2015), fatty acids, and sterols, volatile compounds, glycosides and polyphenols (Erdogan-Orhan and Kartal, 2011). Lutein, *α*- and *β*-carotenes are the main carotenoids of apricot fruit (Erdogan-Orhan and Kartal, 2011; Katayama et al., 2011). *β*-carotene accounts for 60−70% of the total carotenoid content (Dragovic-Uzelac et al., 2007). Apricot is also a rich source of the carotenoid’s precursors phytoene and phytofluene (Biehler et al., 2011). These carotenoids have been largely ignored in the context of agro-food and health and are the major abundant dietary carotenoids (Mapelli-Brahm et al., 2017). Violaxanthin is also present in apricot (Katayama et al., 1971). Polyphenols found in apricot include catechin, hydroxycinnamic acids (chlorogenic, neochlorogenic acids, *p*-coumaric, **Table 1**), epicatechin, epigallocatechin, kaempferol-3-rutinoside, quercetin-3-glucosides, and rutin (**Table 1**, Dragovic-Uzelac et al., 2007; Campbell et al., 2013). Recently, some other minor phenolic compounds were identified, such as hyperoside, narcissin, and naringenin (Di Vaio et al., 2019). Among anthocyanins, cyanidin-3-O-rutinoside (the most abundant, **Table 1**), cyanidin-3-O-glucoside and peonidin-3-O-rutinoside have been detected in some accessions (Bureau et al., 2009). *p*-Coumaric acid has strong antioxidant potential and besides, it has been identified in apricot fruit, and its amounts and those of coumaroyl hexose are very low (Dragovic-Uzelac et al., 2005). Chlorogenic (caffeoyl quinic acid) is an abundant polyphenol in apricot (Dragovic-Uzelac et al., 2005). Caffeic acid has also been detected (Campbell et al., 2013).

About 200 different volatile compounds have been described in apricots (El Hadi et al., 2013). The main volatile phytochemicals include aldehydes, alcohols, ketones, esters, terpenes, and hydrocarbons. The most abundant are hexanal, (E)-2-hexenal, linalool, 1-hexanol, ethyl octanoate, and hexyl acetate (**Table 2**). These volatiles are recognized as major contributors to apricot scent (Aubert and Chanforan, 2007; González-Agüero et al., 2009). The aldehydes hexanal and (E)-2-hexenal display the higher concentrations and have been shown to decrease during ripening. Terpene compounds (*i.e.*, linalool) and alcohols (*i.e.* 1-hexanol) are less abundant than aldehydes, and decrease with ripening (**Table 2**, González-Agüero et al., 2009).

### Plums

Plums may be good sources of natural antioxidants. Plums contain high amounts of polyphenolic compounds, which include anthocyanins, hydroxycinnamates, flavan 3-ols and flavonols. The most common and predominant are chlorogenic acid, neo-chlorogenic acid, catechin, epicatechin, and quercetin-3-rutinoside (Rutin) (**Table 1**, Kim et al., 2003; Tomas-Barberán et al., 2001). However, differences in contents were found in different species of plum, such as *Prunus domestica*, *Prunus salicina*, and *Prunus cerasifera* (Moscatello et al., 2019). Other flavonol glycosides such as cyanidin-3-glucoside and cyanidin-3-galactoside have also been described. Anthocyanins are found in fresh plums predominantly as rutinoside derivatives, such as cyaniding-3-rutinoside (keracyanin, **Table 2**), cyanidin-3-glucoside (kuromanin), and peonidin 3-rutinoside (Raynal et al., 1989; Kim et al., 2003). It is important to mention that anthocyanins have not been detected in yellow plums (Kim et al., 2003). Although plums are not the richest source of carotenes, they contain neoxanthin, lutein, and violaxanthin (Biehler et al., 2011).

Thirty-six different volatile compounds have been identified in Japanese plums (Lozano et al., 2009). In general, they are grouped in esters and lactones, with hexanal, butyl acetate, (E)-2-hexenal, butyl butyrate, hexyl acetate, linalool (2,6-dimethyl-2,7-octadien-6-ol), *γ*-decalactone and *γ*-dodecalactone being the most abundant. Hexanal, provides the plum-like aroma (Gómez et al., 1993 and references therein). Levels of some representative volatiles are shown in **Table 2**.

Not only the number of identified volatiles is shorter in plum than in apricots and peach, but also, they have been found in lower amounts (Gómez et al., 1993). Particularly, while the concentrations of C_6_ compounds (hexanal, (E)-2-hexenal, hexanol, (Z)-3-hexen-1-ol), and their esters are higher in plums than in apricots, apricots are richer in aromatic compounds (**Table 2**). Hydrocarbons were more frequent in plums than in apricots (Gómez et al., 1993).

### Almond Kernels

Bitter almonds have important quantities (3–9%) of amygdalin, which releases hydrocyanic acid and benzaldehyde upon enzymatic hydrolysis (Wirthensohn et al., 2008) and are mainly used in the production of flavor extracts. In turn, sweet almond is consumed as a whole nut, blanched or peeled in the form of a healthy snack or ingredient (Yada et al., 2011). Polyphenols contribute to color and to the moderate astringency. The most predominant polyphenols are proanthocyanidins, hydrolysable tannins, and flavonoids. The amount of some representative polyphenols in different *P. armeniaca* cultivars is presented in **Table 1**.

Almond major proanthocyanidins include epicatechin and catechin. Epiafzelechin is a minor proanthocyanidin. Tannins render gallotannins and ellagitannins after hydrolysis. More than 25 flavonoids have been described in almonds including anthocyanidins (derived from the hydrolysis of proanthocyanidins), flavan-3-ols (catechin, dihydrokaempferol, dihydroquercetin, epicatechin, epicatechin gallate, epicatechin glycoside, and gallocatechin gallate), flavan-3-ols (dihydrokaempferol, catechin, and epicatechin), flavonols (isorhamnetin, kaempferol, quercetin and their 3-O-glucosides, galactosides, and rutinosides), and flavanones (eriodictyol, naringenin, and 7-O-glucosides), and a biflavone. Flavonols are the most abundant flavonoid class in almond. Phenolic acids, lignans, isoflavones, and stilbenes are less represented polyphenols (Bolling, 2017). Almonds are rich in unsaturated lipids and in *α*-, *δ*-, *β*-, and *γ*-tocopherol (Franklin et al., 2017).

Volatiles isolated from almond include more than 20 compounds such as alkylfuranones, n-alkanes, cyclopentadiene and aromatic compounds, such as benzaldehyde, methyl phenol, benzyl alcohol, and some alkylbenzenes. Among the dominant components is benzaldehyde, which has been reported as a predominant volatile of kernel oils from the botanical family Rosaceae and is associated with a marzipan-like flavor, benzyl alcohol, and methyl benzene (**Table 2**). Octane, n-tridecane, n-tetradecane, and hexadecane are the n-alkanes identified (Picuric-Jovanovic and Milovanovic, 1993).

On the other hand, almond skin is a source of bioactive polyphenols and thus of antioxidant activity. About 30 phenolic compounds have been identified which include flavan-3-ols (the more abundant comprising proanthocyanidins, catechin, epicatechin), flavonol glycosides (kaempferol-3-O-rutinoside, kaempferol-3-O-glucoside, isorhamnetin-3-O-rutinoside, isorhamnetin-3-O-glucoside, and quercetin-3-O-glucoside), hydroxybenzoic acids (*p*-hydroxybenzoic acid, vanillic acid, and protocatechuic acid) and aldehydes (protocatechuic aldehyde), flavonol aglycones (kaempferol, quercetin, and isorhamnetin), flavanone glycosides (naringenin-7-O-glucoside and eriodictyol-7-O-glucoside), flavanone aglycones (naringenin and eriodictyol), hydroxycinnamic acids (trans-*p*-coumaric acid and chlorogenic acid), and dihydroflavonol aglycones (dihydroquercetin) (**Table 1**, Garrido et al., 2008).

### Sweet Cherries

Cherries are rich in phenolic compounds, mainly represented by hydroxycinnamates, anthocyanins, flavan-3-ols and flavonols (**Table 1**, Gonçalves et al., 2004).

The main anthocyanins in cherries are cyaniding-3-glucoside, cyanidin-3-rutinoside (Gonçalves et al., 2007; Goulas et al., 2015). While pelargonidin-3-O-rutinoside (Mozetic et al., 2002) and peonidin-3-O-rutinoside occur at low levels in some cultivars (Gonçalves et al., 2007), they were not detected in others (Goulas et al., 2015). Cyanidin-3-rutinoside represents 90% of the total anthocyanin content (Usenik et al., 2008). Sweet cherries are a good source of phenolic acids such as hydroxycinnamic acid derivatives (neochlorogenic acid, *p*-coumaroyl quinic acid and chlorogenic acid) (Liu et al., 2011) Flavonoids detected in sweet cherries include catechin, epicatechin, rutin, quercetin, quercetin-3-rutinoside, quercetin derivative, and kaempferol derivative (Chockchaisawasdee et al., 2016). Fingerprinting conducted by Goulas et al. (2015) revealed that in sweet cherries the levels of hydroxycinnamates are higher than those of flavonoids. Fresh sweet cherry fruit volatiles include alcohols, aldehydes, ketones, hydrocarbons/terpenes and esters, with aldehydes, alcohols, and esters being the most represented (**Table 2**). Hexanal, (E)-2-hexenal, benzaldehyde, (E)-2-hexen-1-o1, ethyl acetate, and hexanoic acid ethyl ester give the typical sweet cherry scent.

## Pre- and Postharvest Treatments Affecting the Phytochemical Biosynthesis and Composition in Stone Fruit

Apart from the studies related to transcription factors involved in the regulation of secondary organic compounds in stone fruit, studies on peach, cherry, and apricot reveal that the composition and concentration of the different phytochemicals vary among the cultivars (Tomas-Barberan et al., 2001; Gil et al., 2002; Cantín et al., 2009; Di Matteo et al., 2017). However, still very few studies have investigated the profiles and content of phenolic and volatiles in several cultivars (Di Vaio et al., 2008; Wang et al., 2009; Di Vaio et al., 2015; Di Matteo et al., 2017). Moreover, the molecular basis for the differences in phytochemical content among varieties has not been investigated in detail yet.

In addition, other factors, like the rootstock (Remorini et al., 2008), the water supply (Tavarini et al., 2011), the growing region, climatic conditions, plant density, nutrient management and agronomic practices (Dragovic-Uzelac et al., 2007; Buendía et al., 2008; Álvarez-Fernández et al., 2011), the state of maturation and various factors in the postharvest stage, and handling (Gil et al., 2002; Dragovic-Uzelac et al., 2007; Tavarini et al., 2011; Scordino et al., 2012; Dabbou et al., 2016; Jia et al., 2009) influence fruit quality and the content of secondary organic compounds and thus consumers acceptance (Minas et al., 2018). Regarding the geographic growing regions, the effect of altitude in the skin pigmentation of peach fruit has been studied. Orchards from higher altitudes showed higher content of total phenols, flavonoids, carotenoids, and anthocyanins in comparison with those grown at lower altitudes (Karagiannis et al., 2016). The content of other metabolites such as quercetin-3-glucoside in apricot does not vary with the ripening stage (Campbell et al., 2013); however, it has been shown that fruit:leaf ratio has an impact on the phytochemical composition of the fruit. For example, fruit thinning, the adjustment of the fruit number of the tree, during pit hardening of apricot improved the phytochemical (total phenolics) content in some cultivars (Roussos et al., 2011). Other practices such as nitrogenous fertilization also affects the phenolic, flavonoid, and anthocyanin contents as it is the case of peach fruit (Vashisth et al., 2017). Depending on the fruit species and the phytochemical considered, the level of secondary organic metabolites might vary upon developmental and ripening stage. For example, in apricots, while the content of some phenolic compounds was constant during ripening, the amounts of others varied (Dragovic-Uzelac et al., 2007). In addition, the content of carotenoids increased during ripening in three different cultivars tested at two different locations (Dragovic-Uzelac et al., 2007). In consequence, the harvest time might affect the health-beneficial properties of the fruit. In this respect, research conducted in plums shows the evolution of the content of different secondary organic metabolites such as carotenoids, total phenolics, anthocyanins during development and ripening on tree of different cultivars (Díaz-Mula et al., 2008; Jiang et al., 2019). Nevertheless, these works showed contradictory results *i.e.*, while Díaz-Mula et al. (2008) described a decrease in phenolics over development of plum; Jiang et al. (2019) showed an increase of phenolic compounds. Later, Moscatello et al. (2019) studying the evolution of sugars, organic acids, and bioactive compounds over development, ripening and overripening, showed that metabolite content is influenced by dilution effects due to the expansion of the fruit. Thus, while phenolics exhibit a net decrease when expressed in terms of fresh or dry weight basis, the net content per fruit increases. Therefore, during plum fruit growth and development there is a net increase in the amount of these bioactive compounds due to synthesis.

On the other hand, the application of different postharvest abiotic stress in horticultural crops has proven to be a simple and effective technology to induce the accumulation of secondary metabolites with a wide range of applications in dietary supplements, functional foods, pharmaceutical markets, cosmetics and agrochemicals. Thus, the use and/or generation of wounds, ultraviolet light, modified atmospheres and phytohormones (ethylene and methyl jasmonate, *etc.*) in fresh fruits and vegetables induces the accumulation of antioxidants (Cisneros-Zevallos, 2003; Jacobo-Velázquez and Cisneros- Zevallos, 2012). More recently, emerging technologies such as ultrasound have been used to generate abiotic stress to induce the accumulation of phenolic compounds (Jacobo-Velázquez and Cisneros- Zevallos, 2018). Particularly in peaches, it was found that the effectiveness of the treatment with UV-B in the modulation of the concentration of phenolic compounds and the expression of the genes involved in the synthesis of phenylpropanoids was genotype-dependent (Scattino et al., 2014; Zhao et al., 2017). UV-A and UV-B treatment increased the content of flavonoids, ascorbate, and cyanidin-3-O-glucoside, and thus, it increased the antioxidant activity of treated peach (Sgherri et al., 2015). In line with these results, bagging also affects the accumulation of nutraceutic compounds. Yellow paper prevents the penetration of blue and UV light resulting in poor coloration of the skin (Liu et al., 2015). In contrast, white non-woven polypropylene allows the accumulation of anthocyanin in the skin. Liu et al. (2015) showed increased expression of the TFs *PpMYB* 10.1, *PpMYB* 10.2, and *PpMYB* 10.3 and their partners *PpbHLH3* and *PpWD40-1* in peach covered with white non-woven polypropylene with respect to fruit covered with yellow paper. Enhanced expression of regulatory genes resulted in higher levels of *PpCHS*, *PpDFR*, and *PpUFGT* in peach bagged using non-woven polypropylene. UV-C treatment during the postharvest storage is also effective in inducing anthocyanin accumulation in cold stored peach (Zhou et al., 2020). In sweet cherries, UV-C applied to fruit after harvest and then cold stored also resulted in an increase in anthocyanins, flavonoids, and total phenolics (Michailidis et al., 2019). Temperature during the postharvest also affects total phenolics and anthocyanin levels. While cold storage at 1–2°C decreases total phenolic content (with neochlorogenic and p-coumaroylquinic acids been the most abundant) in four cultivars of sweet cherries harvested at ripe stage, storage at 15 ± 5°C increases them. In contrast, anthocyanin increased up to fivefold at both temperatures in both ripe and in partially ripe cherries (Gonçalves et al., 2004). On the other hand, after 15 days of fruit storage at 1°C, Esti et al. (2002), also testing two cultivars harvested at commercial maturity indicated a considerable decrease in anthocyanin content. With respect to apricot, heat stress was effective to raise the antioxidant capacity (Madrau et al., 2009).

Preharvest and postharvest oxalic acid (OA) treatment has been successfully applied to stone fruits to increase their nutraceutical properties. In sweet cherries, treatments with OA during development (Martinez-Espla et al., 2014; Martinez-Espla et al., 2019) or during the postharvest followed by 50-day cold storage (Valero et al., 2011) were effective to induce total phenolics, anthocyanins, and antioxidant activity. In peach, OA application 15 days before harvest improved total flavonoids, phenolics, and antioxidant activity in cold stored peach for 28 days (Razavi and Hajiloub, 2016). In addition, in plum (*Prunus salicina* Lindl. ‘Black Splendor’), OA applied as a foliar spray during fruit development successfully increased total phenolics and total antioxidant activity in harvested fruit and after cold storage (35 days at 2°C + 1 day at 20°C, Serrano et al., 2018).

Postharvest treatment on several Prunus fruit with methylcyclopropene (1-MCP), an inhibithor of ethylene perception (Sisler and Serek, 1997), provoked a delay of color development (Valero et al., 2005). Application of 1-MCP resulted in a negative effect on the expression of genes involved in the biosythentic pathway of carotenoids (Marty et al., 2005; Ziliotto et al., 2008) and anthocyanins (Niu et al., 2017) thus, supporting the regulatory role of ethylene in the accumulation of carotenoids and anthocyanins in this species.

## Future Perspectives for Improving Type and Levels of Secondary Phytochemicals in Stone Fruit

Despite the well-known beneficial effects of fruits and vegetables in human health, consumption is still low. Therefore, great efforts should be made to increase the level of health promoting compounds in plant foods by both molecular and non-molecular methods. During the last years, important advances with regard the regulation of secondary metabolism, together with the coordination with primary metabolism, have been achieved in model species such as arabidopsis and tomato. Modification of the expression of individual TFs modifies the levels of enzymes involved not only in secondary but also in primary metabolism suggesting supercoordinated gene expression networks between primary and secondary metabolisms (Aharoni and Galili, 2011). In this respect, it has been proposed that the manipulation of primary metabolism (source of precursors) is a promissory strategy to alter secondary metabolite content (Pott et al., 2019).

In stone fruit, even though in the recent years there has been an increase in genetic (genetic linkage maps and markers associated to quality traits) and omics (genome sequences, transcriptomes, metabolomes, proteomes and volatilomes) resources (Aranzana et al., 2019), there is still limited knowledge about the main factors regulating the accumulation of secondary organic metabolites. Upcoming research of pre- and posthavrest treatements on phytochemical composition should also consider the differences in development and ripening stages of the fruit. More studies should be performed in the future because, as stated in the present review, there is large evidence that stone fruits are rich in secondary organic metabolites, and thus, they may become biofactories of health promoting compounds. The manipulation of primary metabolism to alter secondary metabolite content (Pott et al., 2019) is also a strategy to be considered in stone fruits.

Besides, several studies have highlighted the importance of epigenetic influence in plant ontogenesis, flowering time, heterosis, and fruit ripening process in other species. Much of the information regarding genetic control and epigenetic regulation has been obtained in tomato. The main actors (TF) involved in the transition from fruit growth to ripening have been identified (Giovannoni, 2007), and epigenetic regulation of the targets of these master regulators has also been described in tomato (Giovannoni et al., 2017). Regarding postharvest, epigenetic regulation of the senescence process has been explored in tomato, strawberry, and citrus (Farinati et al., 2017). In comparison, in stone fruits information is scarce, but it is starting to emerge (Fresnedo-Ramírez et al., 2017; Ma et al., 2018). Future studies are really needed and will help in the elucidation of epigenomic dynamics and the epigenetic mechanisms in stone fruit, which may also control secondary organic metabolite production and its relationship with stress condition exposures.

Finally, apart from a deeper knowledge about the molecular mechanisms related to the regulation of the levels of secondary organic compounds in stone fruits, the phytochemical content of the large number of different stone fruit varieties should be deeply characterized. Besides, the molecular mechanisms underlying the differences in phytochemical contents among varieties of the same stone fruit species should be identified. These results could be used in breeding programs to enhance nutritional value of stone fruits, which may aid, along with improved pre- and postharvest handling strategies, in the enlargement of the health promoting compounds of stone fruit for consumers’ benefits in the years to come.

## Author Contributions

All authors contributed to the article and approved the submitted version. However, MD and ML had a major role in the design and writing of the article.

## Funding

ML and MD acknowledge funding from CONICET and ANPCYT.

## Conflict of Interest

The authors declare that the research was conducted in the absence of any commercial or financial relationships that could be construed as a potential conflict of interest.
